# The Invisible Costs of Cancer Treatment: Quantifying Non‐Medical Economic Consequences for Cancer Survivors Undergoing Systemic and Radiation Therapies

**DOI:** 10.1002/cam4.71309

**Published:** 2025-10-21

**Authors:** Md. Shahjalal, Padam Kanta Dahal, Md. Parvez Mosharaf, Yifu Chen, Mohoua Azmary, Edward Christopher Dee, Khorshed Alam, Rashidul Alam Mahumud

**Affiliations:** ^1^ Department of Public Health North South University Dhaka Bangladesh; ^2^ Research Rats Dhaka Bangladesh; ^3^ School of Health, Medical and Applied Sciences, Central Queensland University Rockhampton New South Wales Australia; ^4^ Appleton Institute, Physical Activity Research Group Central Queensland University Rockhampton Queensland Australia; ^5^ School of Business, Faculty of Business, Education, Law and Arts University of Southern Queensland Toowoomba Queensland Australia; ^6^ Centre for Health Research University of Southern Queensland Toowoomba Queensland Australia; ^7^ Bioinformatics Lab, Department of Statistics University of Rajshahi Rajshahi Bangladesh; ^8^ School of Public Health, Faculty of Medicine and Health The University of Sydney Camperdown New South Wales Australia; ^9^ School of Public Health, Faculty of Health and Education Torrens University Surry Hills New South Wales Australia; ^10^ Department of Radiation Oncology Memorial Sloan Kettering Cancer Center New York New York USA; ^11^ NHMRC Clinical Trials Centre, Faculty of Medicine and Health The University of Sydney Camperdown New South Wales Australia

**Keywords:** cancer survivors, necessities, non‐medical economic consequences, schooling disruption, social participation, systemic and radiation therapy

## Abstract

**Background:**

The non‐medical economic consequences of cancer on individuals and families in low‐ and middle‐income countries, including Bangladesh, remain poorly quantified. This study measures the non‐medical economic consequences of cancer survivors and their families, including sacrifices in essential family consumption, social participation and family members' schooling.

**Methods:**

We conducted a cross‐sectional survey between January and May 2022, involving 607 adult patients receiving cancer treatment at two tertiary specialised cancer hospitals in Bangladesh. Participants reported any occurrence of non‐medical economic consequences: reduction in essential goods (REG) consumption, avoidance of social events (ASE) and cessation of schooling (CS) of family members. We used a multivariable logistic regression model to assess the associations between each non‐medical economic consequence and clinical and socioeconomic factors. Additionally, we fitted a Poisson regression to model the count of non‐medical economic consequence events as a function of disease stage and relevant covariates.

**Results:**

Overall, 39% of patients reported REG, 36% ASE, and 38% CS. After adjusting for co‐variates, advanced cancer (stage IV) was a significant predictor of non‐medical economic consequence (REG: OR = 5.58, 95% CI 0.99–31.44, *p* = 0.051; ASE: 11.35, 1.81–71.07, *p* = 0.009; SS: 101.56, 2.47–4173.90, *p* = 0.015) compared with early cancer (stage I). Compared with the highest‐income families, patients from low‐ and lower‐middle‐income families had significantly higher odds of experiencing all outcomes (REG: OR = 7.57, 3.01–19.06, *p* < 0.001 and 7.23, 1.81–28.89, *p* = 0.005; ASE: 10.21, 3.81–27.36, *p* < 0.001 and 17.99, 4.26–75.96, *p* < 0.001; CS: 11.37, 3.23–40.00, *p* < 0.001 and 12.58, 1.90–83.19, *p* = 0.009). Employed patients were also at markedly higher risk, with substantially increased odds of REG (18.27 times), ASE (41.01 times) and CS (72.34 times) compared with non‐employed patients (all *p* < 0.01).

**Conclusion:**

Cancer treatment in Bangladesh imposes substantial and inequitable non‐medical economic consequences, disproportionately affecting patients with advanced‐stage cancer, lower‐income families and those in active employment. Policymakers may prioritise strengthening early cancer detection programs, expanding targeted financial protection schemes and introducing workplace and educational support mechanisms to safeguard family welfare and promote more equitable outcomes during cancer treatment.

**Abbreviations**
ASE: avoidance of social events
CS: cessation of schooling
LMICs: low‐ and middle‐income countries
REG: reduction in essential goods

## 1 Introduction

Cancer is a growing global health crisis, and its impact extends far beyond the clinic, imposing an unbearable economic consequence on patients and their families, particularly in low‐ and middle‐income countries (LMICs) [1], including Bangladesh, a South Asian country of more than 170 million people [2]. The global economic cost of cancer over the next three decades is estimated at US $25 trillion (approximately 0.55% of annual world gross domestic product), encompassing direct medical costs, productivity losses, unemployment and capital investment reductions [3]. While high‐income countries account for the largest absolute expenditures, LMICs often experience a more severe financial burden due to a greater reliance on out‐of‐pocket (OOP) payments for increasingly costly cancer therapies and medical care [1, 4].

Beyond the direct costs of healthcare, cancer imposes indirect financial hardships on patients and their families [5, 6]. A growing body of evidence shows a frequent co‐occurrence of financial distress, social isolation, and educational disruption follows a cancer diagnosis [6, 7, 8, 9, 10]. Patients with cancer may be forced to make trade‐offs in allocating limited household financial resources between their own medical needs and their family's basic needs such as food, clothing, housing, social events, healthcare, and even children's education [6, 9, 10, 11]. A qualitative study reported substantial social consequences, such as reduced social interaction, which can lead to additional distress for people with cancer and their families [6]. These sacrifices can severely compromise quality of life, aggravate psychological distress and widen existing socioeconomic disparities.

Bangladesh is a striking example of these challenges. In 2022, the country recorded an estimated 167,256 new cancer cases and 116,598 deaths, with corresponding incidence and mortality rates of 105.6 and 74.7 per 100,000 population, respectively [12]. Studies within Bangladesh have begun to document the financial strain of cancer, with one recent study reporting that 34% of cancer survivors experienced extreme economic burden due to treatment costs [2]. Another study has suggested disparities in care quality and a high prevalence of psychological morbidity impacting health‐related quality of life among Bangladeshi cancer patients [13]. Given that OOP expenditure is the dominant mode of cancer care financing in Bangladesh [14], it is unsurprising that over a third of survivors report severe economic hardship [2]. While the direct financial burden has received some attention, the critical question of how treatment costs compel families to make sacrifices in fundamental aspects of life, such as essential goods, social participation and education, remains largely unquantified.

To address this significant gap in understanding the broader economic consequences of cancer treatment in this vulnerable setting, this study intends to investigate the non‐medical economic consequences of cancer treatment among Bangladeshi survivors. Specifically, this research aims to answer the following research questions: (1) What proportion of patients experienced a reduction in spending on daily essentials, avoidance of social events and interruptions to schooling for children or other family members due to cancer treatment costs? (2) What clinical and sociodemographic factors are associated with each of these non‐medical economic consequences among Bangladeshi cancer survivors?

Cancer‐related financial hardship can be divided into three domains: material (e.g., debt, OOP costs), psychological (e.g., financial and disease burdens) and behavioural (e.g., coping actions like skipping care or cutting spending) [15, 16]. While previous studies in Bangladesh have measured material hardship [2, 14, 17], little is known about the specific behavioural strategies families use. This study fills that gap by examining the behavioural domain, measuring how families sacrifice essentials, social activities and education—decisions that directly affect family well‐being and human capital. These findings may guide cancer policy development in Bangladesh and similar low‐resource settings.

### 1.1 Theoretical Foundation

This study draws on household resource‐allocation and consumption‐smoothing theories to explain how cancer treatment shocks translate into non‐medical economic consequences (e.g., reductions in consumption of essential goods (REG), avoidance of social events (ASE) and cessation of schooling (CS) among family members). In standard microeconomic models, families maximise utility by allocating limited resources across competing needs; however, large health expenses can exhaust both income and savings, forcing reductions in non‐medical consumption (e.g., essential goods) when credit markets are imperfect or inaccessible [18]. The lifecycle and permanent‐income hypotheses further suggest that, in the absence of comprehensive social risk pooling mechanisms (i.e., insurance), medical shocks lead to sharp declines in both durable and non‐durable consumption rather than temporary reallocation alone [19]. In the context of cancer, these mechanisms are evident as REG consumption, ASE and CS among children, strategies through which families attempt to shield the financial strain of healthcare but incur broader welfare losses.

These microeconomic frameworks are supported by the widely used three‐domain model of financial hardship, which helps us understand the patient's perspective [15, 16, 20]. Our study's outcomes, REG, ASE and CS, directly measure the behavioural domain of this model. They demonstrate the tangible, often severe, actions families undertake when cancer costs exceed their resources. By using this framework, we link economic theories of resource allocation to the real‐life experiences of families, reflecting the ‘capability deprivations’ described by Amartya Sen [21].

Complementing these economic models, Amartya Sen's capability approach highlights that well‐being depends not only on financial resources but on the real freedoms or ‘capabilities’—individuals possess to lead lives they value [21]. Health shocks that compel families to forgo basic goods, social participation and education represent capability deprivations extending beyond measurable financial toxicity. By situating our analysis within these complementary frameworks, we underscore that the invisible costs of cancer treatment encompass both immediate economic sacrifices and longer‐term erosions of social and human capital.

## 2 Methods

### 2.1 Study Design and Settings

We conducted a cross‐sectional survey between January and May 2022 at the National Institute of Cancer Research and Hospital (NICRH) and the Ahsania Mission Cancer and General Hospital (AMCGH) in Bangladesh. The NICRH is the country's only government‐funded tertiary cancer centre, and AMCGH is the country's largest private tertiary cancer treatment facility, located in the capital city of Dhaka. These facilities offer comprehensive diagnostic and treatment services, and draw patients from diverse socioeconomic and demographic backgrounds.

### 2.2 Participants and Survey Procedures

The study population included adults (≥ 18 years) cancer patients who were actively receiving systemic and/or radiation therapy at the outpatient departments of the study hospitals during the survey period. We ensured that the participants had a histologically confirmed diagnosis of any malignancy. We excluded patients with severe physical conditions or major psychiatric illnesses from the survey. We informed all participants that their involvement in the study was completely voluntary, and they provided verbal and written consent (or fingerprints for those who were illiterate) to join the study. A team of trained medical students collected the data after receiving instruction in ethics, privacy, cultural awareness and unbiased interviewing techniques. We conducted a pilot survey with 24 patients to identify and resolve potential issues, interviewed each participant privately and reviewed the data sheets to ensure completeness, accuracy and consistency.

We approached approximately 800 patients, of whom 645 consented and were interviewed (response rate: 80.6%). After excluding 38 cases with incomplete data (< 1% of total participants), 607 complete records remained for analysis.

### 2.3 Outcome Measures

We defined three binary outcome measures to capture the non‐medical economic consequences of cancer treatment. First, a reduction in essential goods consumption was measured by the question: ‘Did you need to cut expenditures on daily necessities (e.g., food, clothing, accommodation or non‐treatment healthcare) due to the costs of your cancer treatment?’ Second, avoidance of social participation was assessed with: ‘Did treatment‐related expenses force you to forgo attending social events (e.g., family gatherings, weddings, restaurant meals or sporting events)?’. Third, interruption of family members' education was determined by: ‘Did treatment costs compel you to stop or interrupt the schooling of your children or other household members?’. Respondents answering ‘yes’ were coded as 1 (indicating the presence of that hardship) and ‘no’ as 0.

To examine the cumulative non‐medical economic consequences, we also constructed a count outcome by summing the three individual indicators for each respondent (range 0–3). This allowed us to model both the occurrence and the magnitude of non‐medical economic consequences arising from cancer treatment expenses.

### 2.4 Clinical Characteristics

We considered the cancer stage a clinical characteristic as it is a crucial criterion for determining the extent and spread of the disease, guiding treatment decisions and influencing prognosis. We obtained information related to cancer stages from the patient's pathology reports and categorised the cancer stages as follows: stage I (early stage or cancer is small and only in one area), stage II (cancer is larger than in stage I), stage III (cancer has grown into nearby tissues or lymph nodes) and stage IV (advanced or metastatic cancer).

### 2.5 Sociodemographic Characteristics

Participants' sociodemographic characteristics included age (in years), sex (male vs. female), height (in inches), weight (in kilograms), marital status (never married, widowed/divorced or married) and residence (urban vs. rural). We categorised education into five levels: no education, primary (1–5 years), secondary (6–10 years), higher secondary (10–12 years) or tertiary (> 12 years of schooling). Occupational status included unemployed, employed, business, informal worker, housewife, student or retirees/others. We grouped monthly family income into five quintiles: Q1 (20% lowest quintile: poorest) to Q5 (20% highest quintile: richest).

### 2.6 Statistical Analysis

We first described the study sample using frequencies (*n*) and percentages (%) for all categorical variables. To quantify the association between each non‐medical economic consequence, measured as REG, ASE and CS, and the explanatory variables, we fitted separate multivariable logistic‐regression models. Binary coding of the outcomes (0 = no or 1 = yes) justified the use of a logistic regression model rather than a linear regression model. To examine the cumulative non‐medical economic consequences, we generated a composite count measure by summing the three hardship indicators for each respondent (range 0–3). Given the count distribution and absence of overdispersion, we applied a Poisson regression model to examine the rate of non‐medical economic consequence events as a function of cancer stage and covariates. Variables with *p* value ≤ 0.05 in univariable analyses were entered into the multivariable model to control for confounding. Adjusted odds ratios (AOR) or incidence rate ratios (IRR) with 95% confidence intervals (95% CIs) were reported, and statistical significance was set at two‐sided *p* ≤ 0.05. We performed all statistical analyses using the Stata/SE version 15 (Stata Corp LLC, College Station, TX, USA). We applied a complete‐case analysis principle in this analytical exploration.

## 3 Results

The analytic sample comprised 607 patients, of whom 55% were female and 44% were aged 46–64 years (Table S1). Table 1 shows that non‐medical economic consequences, measured as reduction in essential goods (REG), avoidance of social events (ASE) and cessation of schooling (CS), were highly prevalent among socioeconomically vulnerable groups. For example, 39% of patients in the lowest income quintile reported REG, 36% reported ASE, and 38% reported CS; similar proportions were observed among unemployed individuals and homemakers (40% REG, 38% ASE, 40% CS). Non‐medical economic consequences were most extreme among married patients (REG 89%; ASE 88%; CS 83%) and those living in rural areas (REG 90%; ASE 89%; CS 86%), underscoring how marital status and geographic isolation intersect with these consequences.

**TABLE 1:** Distribution of non‐medical economic consequences according to the patients' characteristics due to cancer treatments.

Variables	No experience with any issue, *n* (%)	Prevalence of REG, *n* (%)	Prevalence of ASE, *n* (%)	Prevalence of CS, *n* (%)
Cancer stage				
Stage I	3 (4.55)	13 (2.97)	14 (3.14)	1 (0.71)
Stage II	37 (56.06)	179 (40.87)	180 (40.36)	46 (32.60)
Stage III	20 (30.30)	161 (36.76)	141 (31.61)	46 (32.62)
Stage IV	6 (9.09)	85 (19.41)	111 (24.89)	48 (34.04)
Income quintile				
Q1 (poorest family)	12 (18.18)	172 (39.27)	160 (35.87)	53 (37.59)
Q2	3 (4.55)	38 (8.68)	48 (10.76)	9 (6.38)
Q3	10 (15.15)	84 (19.18)	83 (18.61)	26 (18.44)
Q4	16 (24.24)	90 (20.55)	95 (21.30)	28 (19.86)
Q5 (richest family)	25 (37.88)	54 (12.33)	60 (13.45)	25 (17.73)
Occupational status				
Unemployed	4 (6.06)	108 (24.66)	122 (27.35)	33 (23.40)
Employed	2 (3.03)	26 (5.94)	34 (7.62)	9 (6.38)
Business	6 (9.09)	26 (5.94)	25 (5.61)	12 (8.51)
Housewife	37 (56.06)	175 (39.95)	171 (38.34)	56 (39.72)
Informal workers	2 (3.03)	38 (8.68)	32 (7.17)	11 (7.80)
Students	3 (4.55)	9 (2.05)	8 (1.79)	4 (2.84)
Others (e.g., retired)	12 (18.18)	56 (12.79)	54 (12.11)	16 (11.35)
Age in years				
18–35	9 (13.64)	66 (15.07)	64 (14.35)	25 (17.73)
36–45	15 (22.73)	93 (21.23)	101 (22.65)	35 (24.82)
46–64	27 (40.91)	187 (42.69)	198 (44.39)	59 (41.84)
> 64	15 (22.73)	92 (21.00)	83 (18.61)	22 (15.60)
Sex				
Male	23 (34.85)	202 (46.12)	201 (45.07)	62 (43.97)
Female	43 (65.15)	236 (53.88)	245 (54.93)	79 (56.03)
Marital status				
Single or never married	3 (4.55)	20 (4.57)	19 (4.26)	7 (4.96)
Married	55 (83.33)	390 (89.04)	393 (88.12)	117 (82.98)
Divorced/separated/widowed	8 (12.12)	28 (6.39)	34 (7.62)	17 (12.06)
Education				
No education	24 (36.36)	216 (49.43)	216 (48.54)	79 (56.03)
Primary	14 (21.21)	111 (25.40)	11 (25.62)	27 (19.15)
Secondary	11 (16.67)	72 (16.48)	68 (15.28)	17 (12.06)
Higher secondary	9 (13.64)	27 (6.18)	27 (6.07)	11 (7.80)
Tertiary education	8 (12.12)	11 (2.52)	20 (4.49)	7 (4.96)
Body mass index (BMI)				
Underweight	3 (4.55)	82 (18.72)	80 (17.94)	20 (14.18)
Healthy weight	42 (63.64)	260 (59.36)	276 (61.88)	93 (65.96)
Overweight	14 (21.21)	63 (14.38)	59 (13.23)	19 (13.48)
Obese	7 (10.61)	33 (7.53)	31 (6.95)	9 (6.38)
Residence				
Rural	53 (80.30)	396 (90.41)	398 (89.24)	121 (85.82)
Urban	13 (19.70)	42 (9.59)	48 (10.76)	20 (14.18)

Abbreviations: %: percentage; ASE, avoiding attending social events; BMI, body mass index; CS, cessation of schooling of family members; *n*, number of patients; Q, quantile; REG, reducing of essential goods.

Multivariable logistic regression (Table 2) identified advanced disease stage, lower family income and active employment as the strongest independent predictors of non‐medical economic consequences. Compared with stage I survivors, stage IV patients had markedly higher odds of REG (OR 5.58; 95% CI 0.99–31.44, *p* = 0.051), ASE (OR 11.35; 95% CI 1.81–71.07, *p* = 0.009) and CS (OR 101.56; 95% CI 2.47–4173.90, *p* = 0.015). A pronounced income gradient was evident: relative to the richest quintile, survivors in the poorest quintile experienced 8‐fold higher odds of REG (OR 7.57; 95% CI 3.01–19.06, *p* < 0.001), 10‐fold higher odds of ASE (OR 10.21; 95% CI 3.81–27.36, *p* < 0.001) and 11‐fold higher odds of CS (OR 11.37; 95% CI 3.23–40.00, *p* < 0.001). Those in the second‐lowest quintile (Q2) faced comparably elevated odds across all outcomes. Employment status amplified risk: employed survivors had 18‐fold greater odds of REG, 41‐fold greater odds of ASE and 72‐fold greater odds of CS compared with retirees (all *p* < 0.01), with similarly elevated but more modest effects for unemployed and informal workers.

**TABLE 2:** Association of non‐medical economic consequences with clinical and socioeconomic factors.

Factors	Model 1 (REG)		Model 2 (ASE)		Model 3 (CS)	
	OR (95% CI)	*p*	OR (95% CI)	*p*	OR (95% CI)	*p*
Cancer stage (Ref = Stage I)						
Stage II	1.03 (0.23, 4.66)	0.966	0.96 (0.20, 4.61)	0.962	4.43 (0.13, 150.25)	0.408
Stage III	1.98 (0.42, 9.45)	0.390	1.75 (0.35, 8.78)	0.498	10.23 (0.29, 366.28)	0.203
Stage IV	5.58 (0.99, 31.44)	0.051	11.35 (1.81,71.07)	0.009	101.56 (2.47, 4173.90)	0.015
Income quintile (Ref = Q5: richest quintile)						
Q1 (poorest quintile)	7.57 (3.01, 19.06)	< 0.001	10.21 (3.81,27.36)	< 0.001	11.37 (3.23, 40.00)	< 0.001
Q2	7.23 (1.81, 28.89)	0.005	17.99 (4.26,75.96)	< 0.001	12.58 (1.90, 83.19)	0.009
Q3	3.90 (1.53, 9.95)	0.004	5.00 (1.86,13.41)	0.001	3.02 (0.86, 10.64)	0.085
Q4	3.10 (1.30, 7.42)	0.011	4.22 (1.68,10.58)	0.002	2.01 (0.60, 6.72)	0.256
Occupation (Ref = Others)						
Unemployed	13.04 (3.07, 55.43)	0.001	24.24 (5.55, 105.94)	< 0.001	33.47 (6.04, 185.51)	< 0.001
Employed	18.27 (2.77, 120.36)	0.003	41.01 (5.87, 286.74)	< 0.001	72.34 (4.73, 1107.50)	0.002
Business	1.61 (0.44, 5.86)	0.469	2.01 (0.52, 7.74)	0.311	5.2 (0.87, 30.98)	0.070
Housewife	1.22 (0.32, 4.73)	0.771	1.44 (0.35, 60.00)	0.616	1.71 (0.32, 9.09)	0.531
Informal workers	3.76 (0.68, 20.86)	0.129	4.85 (0.82, 28.66)	0.082	14.65 (1.68, 127.43)	0.015
Students	0.71 (0.05, 9.75)	0.797	0.76 (0.05, 11.27)	0.844	2.56 (0.07, 94.08)	0.609
Age in years (Ref = 18–35 years)						
36–45	0.61 (0.19, 1.90)	0.389	0.63 (0.20, 2.01)	0.437	0.43 (0.10, 1.82)	0.251
46–64	0.70 (0.23, 2.12)	0.529	0.62 (0.20, 1.89)	0.397	0.36 (0.09, 1.41)	0.141
> 64	0.66 (0.20, 2.21)	0.497	0.55 (0.16, 1.90)	0.345	0.23 (0.05, 1.18)	0.078
Sex (Ref = Male)						
Female	1.41 (0.39, 5.03)	0.598	1.65 (0.45, 6.08)	0.452	2.38 (0.51, 11.26)	0.272
Marital status (Ref = Single or never married)						
Married	1.49 (0.14, 15.50)	0.738	2.26 (0.21, 24.94)	0.504	3.33 (0.07, 153.46)	0.538
Divorced/separated/widowed	0.43 (0.03, 5.49)	0.514	0.73 (0.06, 9.52)	0.807	1.91 (0.03, 116.79)	0.757
Education (Ref = Tertiary education)						
No education	5.37 (1.31, 21.88)	0.019	2.58 (0.67, 9.91)	0.168	3.07 (0.43, 21.73)	0.262
Primary	6.06 (1.49, 24.67)	0.012	2.68 (0.70, 10.24)	0.148	2.30 (0.34, 15.44)	0.392
Secondary	4.90 (1.13, 21.17)	0.033	2.32 (0.56, 9.56)	0.244	1.63 (0.19, 13.57)	0.649
Higher secondary	1.89 (0.43, 8.37)	0.404	0.57 (0.12, 2.60)	0.465	0.75 (0.09, 6.13)	0.788
BMI (Ref = Healthy weight)						
Underweight	4.69 (1.14, 19.36)	0.032	8.37 (1.70, 41.12)	0.009	9.15 (1.14, 73.75)	0.038
Overweight	0.97 (0.44, 2.16)	0.945	0.70 (0.31, 1.61)	0.407	0.34 (0.11, 1.00)	0.051
Obese	0.57 (0.20, 1.61)	0.291	0.49 (0.17, 1.40)	0.181	0.25 (0.06, 1.05)	0.058
Residence (Ref = Rural)						
Urban	0.55 (0.23, 1.30)	0.173	0.69 (0.28, 1.70)	0.415	1.28 (0.40, 4.12)	0.680

*Note:* Variables exhibiting a *p* value ≤ 0.05 in univariable analyses were entered into the multivariable model to control for confounding.

Abbreviations: ASE, avoiding attending social events; BMI, body mass index; CI, confidence interval; CS, cessation of schooling of family members; OR, odds ratio; *p*, the probability value; Q, quantile; Ref, reference category; REG, reducing of essential goods.

Poisson regression (Table 3) confirmed these patterns in the number of hardship events. After adjustment for covariates, stage IV diagnosis was associated with a 44% higher rate of non‐medical economic consequences compared with stage I (IRR 1.44; 95% CI 1.11–1.86; *p* = 0.006). A clear income gradient persisted: survivors in the poorest quintile experienced a 31% higher rate of hardship events (IRR 1.31; 95% CI 1.13–1.52; *p* < 0.001) and those in the second‐poorest quintile a 36% increase (IRR 1.36; 95% CI 1.14–1.62; *p* = 0.001) relative to the wealthiest quintile. Collectively, these findings demonstrate that advanced cancer stage and socioeconomic disadvantage compound the invisible, non‐medical costs borne by survivors and their families.

**TABLE 3:** Factors influencing the number of non‐medical economic consequences among cancer survivors and their families during cancer treatments.

Factors	IRR	95% CI of IRR		*p*
		Lower limit	Upper limit	
Cancer stage (Ref = Stage I)				
Stage II	1.15	0.89	1.49	0.279
Stage III	1.26	0.97	1.63	0.081
Stage IV	1.44	1.11	1.86	0.006
Income quantile (Ref = Q5: richest quintile)				
Q1 (poorest quintile)	1.31	1.13	1.52	< 0.001
Q2	1.36	1.14	1.62	0.001
Q3	1.24	1.06	1.44	0.007
Q4	1.15	0.99	1.34	0.063
Occupation (Ref = Others)				
Unemployed	1.31	1.14	1.50	< 0.001
Employed	1.48	1.22	1.80	< 0.001
Business	1.25	1.02	1.53	0.035
Housewife	1.05	0.89	1.25	0.547
Informal workers	1.32	1.11	1.57	0.002
Students	1.03	0.68	1.54	0.901
Age in years (Ref = 18–35 years)				
36–45 years	0.94	0.83	1.07	0.366
46–64 years	0.88	0.78	1.00	0.045
> 64 years	0.85	0.74	0.98	0.025
Sex (Ref = Male)				
Female	1.06	0.95	1.20	0.296
Marital status (Ref = Single or never married)				
Married	0.89	0.72	1.09	0.243
Divorced/separated/widowed	0.88	0.67	1.15	0.334
Education (Ref = Tertiary)				
No education	1.22	0.92	1.61	0.160
Primary	1.13	0.86	1.48	0.379
Secondary	1.14	0.86	1.49	0.358
Higher secondary	0.91	0.66	1.25	0.552
BMI (Ref = Healthy weight)				
Underweight	1.05	0.96	1.15	0.284
Overweight	0.95	0.83	1.08	0.418
Obese	0.96	0.81	1.15	0.687
Residence (Ref = Rural)				
Urban	0.97	0.84	1.11	0.645

Abbreviations: BMI, body mass index; CI, confidence interval; IRR, incidence rate ratio; *p*, the probability value; Ref, reference group.

## 4 Discussion

Our study explored how patients perceive their cancer diagnosis and its impact on their families, particularly regarding non‐medical economic consequences, reduction in essential goods, avoidance of social events and cessation of schooling of a family member. Our analysis demonstrates a clear socioeconomic gradient in the non‐medical economic consequences of cancer care in Bangladesh. Advanced disease was the single strongest determinant: patients diagnosed at stage IV were substantially more likely than those at stage I to restrict spending on daily necessities, withdraw from social activities and—most alarmingly—interrupt a child's or family member's education. Even after accounting for stage, families in the low‐ and lower‐middle‐income quintiles remained at markedly elevated risk of all three hardships, suggesting that limited financial reserves magnify the indirect costs of treatment. Finally, labour‐force participation emerged as a critical modifier: working‐age patients, especially those in insecure or informal employment, exhibited far greater odds of sacrificing essential goods, social life and schooling than retirees, underscoring the vulnerability of income‐dependent families when illness disrupts earning capacity.

What was strikingly evident from this study is that children and/or any family members of cancer patients may suffer detrimental consequences from a cancer diagnosis, as it halts their education or schooling. Similar to our findings, an earlier American study revealed that children living in families with a history of parental cancer were more likely to face challenges in education, such as frequent school absenteeism and extracurricular activities compared to children without a history of parental cancer [9]. These results suggest that the negative associations between children's education and family history of cancer may be caused by the high OOP expenses of cancer treatment rather than the socioeconomic status of the families [9, 11]. If we consider that families with a history of cancer may already have a lower socioeconomic status, this could worsen their situation and significantly impact children's education and overall well‐being [22]. This is particularly alarming in Bangladesh, where the secondary school dropout rate is roughly 36% [23] and school‐age children make up 23% of the total population [24]. In oncology and primary care, clinicians must be equipped to screen for the non‐medical financial hardships faced by cancer patients and the school‐age children in their families, who may risk missing out on necessary education. Hence, the authors advocate for child‐focused interventions to address economic and social protection, targeted educational policies and school support programs for their education and well‐being.

Patients with advanced cancer are often very ill and receive intensive treatments (e.g., systemic therapy, radiotherapy and surgery) [25], and they commonly experience complications, have multiple comorbid conditions, and generally spend more time in various care settings—such as inpatient care, long‐term care and hospice—than the average cancer patients [26, 27]. Not surprisingly, this contributes to the direct cost of advanced cancer treatment, but there are other non‐medical or indirect costs that are potentially problematic for people with cancer and their families [26, 28]. In our study, we found that relative to stage I, a diagnosis at stage IV multiplies the odds of restricting essential goods, withdrawing from social life and—most strikingly, interrupting schooling. This association with disease stage, which indicates treatment intensity and duration, shows how heavily prolonged, complex care burdens household resources. These findings align with international data demonstrating that late‐stage disease amplifies OOP spending, productivity losses and psychosocial strain. For example, a review highlighted the significant OOP expenses associated with later‐stage cancer treatments and their adverse effects on social lives by limiting social gatherings, causing work productivity losses due to absences and unexpected disruptions in children's education [28]. Studies from some comparative settings in Malawi and Southeast Asia report that OOP costs associated with late‐stage cancer result in severe social and economic repercussions [29, 30]. These findings add compelling evidence to the argument for effective early cancer detection policy and financial protection mechanisms for cancer patients and their families in Bangladesh and similarly other resource‐limited settings.

According to our data, patients in the low‐ and lower‐middle‐income quintiles were 7‐ to 18‐times more likely to sacrifice essentials, social events and schooling than their affluent counterparts. In line with this result, a prospective longitudinal study in eight neighbouring countries from South Asia found that patients from low‐income families faced over five times the likelihood of experiencing financial disaster compared to their high‐income counterparts [30]. This financial crisis for cancer care can force families, particularly low‐middle‐income patients, to cut back on essential goods, draw down social resources, and in some cases, forgo medical care [30, 31, 32]. In Bangladesh, a country with limited resources, the lack of universal health coverage and health insurance necessitates that patients bear substantial OOP costs, making patients and their families more vulnerable to financial hardship [2, 14]. To mitigate this burden, providing affordable health insurance schemes and targeted financial support, such as subsidies or cash transfers, can help this demographic address their heightened vulnerability. Furthermore, community‐based initiatives like pooled health funds and integration of financial counselling into oncology care can offer immediate relief.

The regression analysis revealed that working‐age patients, particularly those in informal or precarious employment, faced odds ratios exceeding 40 for social withdrawal and 70 for educational disruption compared with retirees. In line with our findings, previous studies reported that the financial strain can be compounded by the loss of income if patients or their caregivers are unable to work due to the demands of ongoing treatment and care [8, 10, 30]. A family's financial flexibility diminishes with an increase in dependents, complicating the management of escalating medical expenses for working‐aged cancer patients who are unable to work [30, 31]. Where illness interrupts earnings and social protection is minimal, families rapidly deplete savings, accumulate debt and reallocate resources away from food, clothing and school fees [5, 10, 11]. There is a distinct necessity for comprehensive financial support systems, including sickness benefit programs and employer‐sponsored income replacement initiatives, that cater to patients unable to work.

### 4.1 Limitations

This study has some limitations. First, responses to the outcomes measure were self‐reported by the sample. As such, there is likely recall and reporting bias, which may have been influenced by memory issues or social desirability, particularly for sensitive topics like financial status. Second, the cross‐sectional study design captures a snapshot of hardship among patients at various points in their active treatment journey. We did not set a minimum treatment duration for inclusion, meaning our sample is heterogeneous in this regard. While this precludes an analysis of how hardship accumulates over time, the high prevalence of these consequences observed across the entire sample suggests that for many families in Bangladesh, the non‐medical economic shock of cancer treatment is both severe and immediate. Future longitudinal research is essential to track the trajectory of these behavioural hardships from diagnosis through survivorship. Third, we did not collect detailed data on treatment duration or the number of therapy cycles. While cancer stage is an important driver of treatment intensity and duration, which served as a strong proxy in our models, we acknowledge that more specific variables could add nuance. The strong dose–response relationship we found between cancer stage and all three hardship outcomes suggests our models captured the key clinical driver of this burden. Still, we recommend that future prospective studies gather detailed treatment data to separate the effects of disease stage from treatment duration and intensity on family economic well‐being.

### 4.2 Implications of Findings

The findings of this study have important implications for clinical practice and public health policy in Bangladesh and other similar settings. Our findings demonstrate that advanced cancer is linked to increased likelihoods of restricting essential goods, withdrawing from social interactions and, most significantly, disrupting education. This highlights the need to advocate for early cancer detection through screening, and early diagnosis, which may lead to more efficient treatment, slower disease progression, fewer complications, lower disease management costs and better health outcomes.

The second implication is that in settings where patients with limited family incomes face higher OOP costs for cancer care—as in the current Bangladeshi context [14], the family often plays a crucial role in health and healthcare. In such settings, cancer can profoundly affect patients and their families by imposing financial hardship related to non‐medical costs associated with the disease; this underscores the importance of financial assistance or subsidies to safeguard both health and economic well‐being.

The third implication of the findings indicates that financial devastation is frequently worsened by the loss of income experienced by many working‐age patients, which often leads to financial sacrifices and household impoverishment, impacting families [8, 32, 33]. Thus, cancer patients often feel the need to return to work as soon as possible to alleviate the financial burden of their medical expenses [33]. To support their efforts in retaining or returning to employment, it is essential to have adequate paid medical leave, time off for hospital follow‐ups and flexible work arrangements.

Finally, by recognising and prioritising the family‐oriented non‐medical consequences of cancer, particularly regarding children's education, we can create a more resilient and supportive healthcare framework for patients and their families.

## 5 Conclusions

The trajectory of cancer treatments was associated with non‐medical economic consequences, including restrictions on daily living necessities, reduced participation in social events, and interruptions to the schooling of their children or other family members. Patients with advanced cancer, from low‐ and lower‐middle‐income families and of working age, are particularly vulnerable to these socioeconomic crises. Ultimately, the non‐medical economic hardship of cancer must be recognised not only as a considerable burden to individuals but also to families, with lasting consequences over time. In Bangladesh, it is crucial to emphasise the ongoing economic impact of cancer beyond the medical realm, underscoring the need for comprehensive support systems to improve the overall well‐being of patients and their families.

## Author Contributions

M.S. and R.A.M. conceptualised the study and developed the research idea. M.S., M.P.M., Y.C. and M.A. were involved in the data collection and curation process. R.A.M. performed data analysis and interpreted the data. M.S., P.K.D. and R.A.M. prepared the draft manuscript. M.S., E.C.D., K.A. and R.A.M. revised the manuscript. M.S. and R.A.M. coordinated the study and had full access to all of the data in the study and take responsibility for the integrity of the data and the accuracy of the data analysis.

## Ethics Statement

This study complied with the Declaration of Helsinki. Respondents were reassured that all the information collected would be kept strictly confidential and would not be used for anything other than research purposes. The institutional review board at North South University, Bangladesh, approved the study (Ref‐2021/OR‐NSU/IRB/0401).

## Consent

The authors have nothing to report.

## Conflicts of Interest

The authors declare no conflicts of interest.

## Supporting information


**Appendix S1:** Patients' basic characteristics.

## Data Availability

The data that support the findings of this study are available on request from the corresponding author. The data are not publicly available due to privacy or ethical restrictions.
