# Supplementary material for: The Invisible Costs of Cancer Treatment: Quantifying Non‐Medical Economic Consequences for Cancer Survivors Undergoing Systemic and Radiation Therapies
Source: Cancer Med. 2025 Oct 21;14(20):e71309. doi: 10.1002/cam4.71309 (PMC12538818; doi:10.1002/cam4.71309)
Supplement: Supplementary file 1 — Appendix S1: Patients' basic characteristics. [file CAM4-14-e71309-s001.docx]

**Appendix Table**

**Appendix S1:** Patients’ basic characteristics (n=607)

| **Patients’ characteristics** | **Number (%)** |  | **95% CI** |
| --- | --- | --- | --- |
| **Age, years** |  |  |  |
| 18 to 35 | 84(13.84) |  | 11.31, 16.83 |
| 36 to 45 | 130(21.41) |  | 18.33, 24.87 |
| 46 to 65 | 268(44.15) |  | 40.24, 48.14 |
| >65 | 125(20.59) |  | 17.56, 24.00 |
| **Sex** |  |  |  |
| Male | 275(45.30) |  | 41.37, 49.29 |
| Female | 332(54.70) |  | 50.71, 58.63 |
| **Marital status** |  |  |  |
| Single or never married | 24(3.95) |  | 2.66, 5.84 |
| Married | 537(88.47) |  | 85.67, 90.78 |
| Divorced/separated/widowed | 46(7.58) |  | 5.72, 9.98 |
| **Education** |  |  |  |
| No education | 286(47.11) |  | 43.24, 51.19 |
| Primary (years: 1 to 5) | 153(25.20) |  | 21.94, 28.87 |
| Secondary (years: 6 to 10) | 94(15.48) |  | 12.84, 18.62 |
| Higher secondary (years: 11 to 12) | 45(7.43) |  | 5.59, 9.81 |
| Tertiary (years: >12) | 28(4.62) |  | 3.21, 6.62 |
| **Boby Mass Index (BMI)** |  |  |  |
| Underweight: <18.50 | 100(16.47) |  | 13.73,19.65 |
| Healthy weight: 18.50-24.99 | 373(61.44) |  | 57.5, 65.25 |
| Overweight: 25.00-29.99 | 90(14.82) |  | 12.21, 17.89 |
| Obese: 30 or more | 44(7.24) |  | 5.43, 9.61 |
| **Occupation** |  |  |  |
| Unemployed | 140(23.06) |  | 19.88, 26.59 |
| Employed | 39(6.42) |  | 4.73, 8.68 |
| Business | 41(6.75) |  | 5.01, 9.05 |
| Housewife | 248(40.85) |  | 37.00, 44.83 |
| Informal workers# | 40(6.58) |  | 4.87, 8.87 |
| Students | 13(2.14) |  | 1.25, 3.66 |
| Other occupations | 86(14.16) |  | 11.61,17.18 |
| **Income quintile** |  |  |  |
| Q1 (20% lowest: poorest) | 208(34.26) |  | 30.59, 38.14 |
| Q2 | 53(8.73) |  | 6.73, 11.26 |
| Q3 | 111(18.28) |  | 15.40, 21.57 |
| Q4 | 130(21.41) |  | 18.33, 24.87 |
| Q5 (20% highest: richest) | 105(17.29) |  | 14.49, 20.52 |
| **Residence** |  |  |  |
| Rural | 534(87.97) |  | 85.13, 90.33 |
| Urban | 73(12.02) |  | 9.67, 14.87 |

**Abbreviation**: *CI*=confidence interval, *Q*: quintile.

#**Informal workers** include day labour, home-based workers, street vendors and so on.
